# No escape: The influence of substrate sodium on plant growth and tissue sodium responses

**DOI:** 10.1002/ece3.8138

**Published:** 2021-09-23

**Authors:** Luis Y. Santiago‐Rosario, Kyle E. Harms, Bret D. Elderd, Pamela B. Hart, Maheshi Dassanayake

**Affiliations:** ^1^ Department of Biological Sciences Louisiana State University Baton Rouge Louisiana USA

**Keywords:** biomass accumulation, fitness, halophytes, model selection, plant growth, plant salt stress responses, sodium, sodium accumulation

## Abstract

1. As an essential micronutrient for many organisms, sodium plays an important role in ecological and evolutionary dynamics. Although plants mediate trophic fluxes of sodium, from substrates to higher trophic levels, relatively little comparative research has been published about plant growth and sodium accumulation in response to variation in substrate sodium. Accordingly, we carried out a systematic review of plants' responses to variation in substrate sodium concentrations.
2. We compared biomass and tissue‐sodium accumulation among 107 cultivars or populations (67 species in 20 plant families), broadly expanding beyond the agricultural and model taxa for which several generalizations previously had been made. We hypothesized a priori response models for each population's growth and sodium accumulation as a function of increasing substrate NaCl and used Bayesian Information Criterion to choose the best model. Additionally, using a phylogenetic signal analysis, we tested for phylogenetic patterning of responses across taxa.
3. The influence of substrate sodium on growth differed across taxa, with most populations experiencing detrimental effects at high concentrations. Irrespective of growth responses, tissue sodium concentrations for most taxa increased as sodium concentration in the substrate increased. We found no strong associations between the type of growth response and the type of sodium accumulation response across taxa. Although experiments often fail to test plants across a sufficiently broad range of substrate salinities, non‐crop species tended toward higher sodium tolerance than domesticated species. Moreover, some phylogenetic conservatism was apparent, in that evolutionary history helped predict the distribution of total‐plant growth responses across the phylogeny, but not sodium accumulation responses.
4. Our study reveals that saltier plants in saltier soils proves to be a broadly general pattern for sodium across plant taxa. Regardless of growth responses, sodium accumulation mostly followed an increasing trend as substrate sodium levels increased.

## Graphical abstract

In our systematic review, we examined how plants' growth and tissue‐sodium accumulation responds to variation in substrate sodium concentration. The influence of substrate sodium on growth differed across taxa, with a degree of phylogenetic conservatism. Even so, most populations experienced detrimental effects at high concentrations, and the pattern of saltier plants in saltier soils appears to apply broadly across taxa.

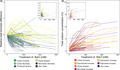

## 1 INTRODUCTION

Plants are key conduits in many, especially terrestrial, biogeochemical cycles (Elser & Bennett, 2011; Farago, 1995; Neubauer et al., 2005; Yuan & Chen, 2015). As intermediaries between soils and higher trophic levels, they often control, limit, or enhance the availability of elements to consumers. Plant phytochemistry varies substantially in elemental composition, stoichiometry, and concentration of essential micronutrients for animals and decomposers (Farago, 1995; Sterner & Elser, 2002). Hunter (2016) envisioned the geographic patterning of phytochemistry as the phytochemical landscape. The phytochemical landscape of micronutrients has considerable effects on plant‐herbivore interactions, as well as community and ecosystem dynamics across landscapes that vary in soils, climate, etc. (Clay et al., 2014; Kaspari et al., 2008; Moore et al., 2010). Nonetheless, the composition, formation, and intermediary function of the phytochemical landscape remains poorly characterized and understood (Hunter, 2016), especially for certain elements such as sodium (Kaspari, 2020).

Sodium is the seventh most abundant element in the Earth's crust (Kaspari, 2020). However, its presence in terrestrial ecosystems is highly heterogeneous, but spatially correlated with xeric conditions, certain geological formations and proximity to a marine coast or source of marine aerosols (Kaspari, 2020; Martin et al., 2010; Smith, 2013; Stallard & Edmond, 1981). Sodium is unusual as a nutrient for life because although it is a nonessential element for most plants, it is a key and essential element for animals and decomposers (Kaspari, 2020). Although sodium requirements vary among organisms, the availability and intake of sodium are tightly linked to organismal performance across ecosystems and form fundamental components of ecological and evolutionary dynamics (Baxter & Dilkes, 2012; Kaspari et al., 2009; Sterner & Elser, 2002).

Plant populations and communities are exposed to a wide range of saline substrates across terrestrial landscapes. Many plants actively avoid or limit sodium intake, and most plants tolerate sodium in soils to remarkably high levels (at millimolar levels) before they show signs of growth defects compared to many other nonessential or toxic cations such as lithium or many heavy metals that induce toxicity symptoms at micromolar levels (Nawaz et al., 2017; Pantha & Dassanayake, 2020; Shahzad et al., 2016; Vithanage et al., 2019; van Zelm et al., 2020). Most plants can tolerate or can be acclimated to survive up to 200 mM NaCl in their growth media, but those plants that can complete their life cycles at salinity levels higher than 200 mM NaCl are generally identified as halophytes (Cheeseman, 2015; Flowers et al., 1986, 2010). Unlike most plants, many halophytes need sodium to thrive and suffer growth defects under limited sodium (Bose et al., 2017; Wang et al., 2012). However, only about 1% of the global flora are considered halophytes; they are distributed in multiple plant clades that reflect their convergent evolution to saline environments (Flowers & Colmer, 2008).

Even though most plants do not need sodium, they cannot necessarily avoid it, nor escape having to cope with it. As sodium concentration increases in the substrate, its concentration in plant tissue also generally increases, and in turn affects plant fitness, especially in plants highly sensitive to salt stress (Greenway & Munns, 1980; Pantha & Dassanayake, 2020; Yang & Guo, 2018; Zhu, 2001). With increasing sodium, plants have been shown to decrease biomass accumulation; increase osmotic, oxidative, and ionic stress responses; and arrest growth due to changes in cellular biochemistry (Maathuis, 2014; Zhao et al., 2020). Furthermore, variation in soil concentration of sodium salts has direct links to variation in foliar sodium, which in turn influences plant‐herbivore interactions and higher trophic‐level performance (Bravo, Harms, & Emmons, 2010, 2012; Cheeseman, 2015; Kaspari, 2020; Kaspari et al., 2014; Snell‐Rood et al., 2014).

Decades of physiological, biochemical, and genetic studies have contributed to our current understanding of how plants respond to salt stress. Even so, these studies have primarily targeted salt stress‐sensitive model plants such as *Arabidopsis*, salt‐sensitive crops, or extremely tolerant halophytes. For example, most crops or *Arabidopsis* ecotypes will show signs of salt‐stress at 100 mM NaCl (0.58%) treatments, whereas some halophytes can survive salinities exceeding seawater strengths (3.5%) (Debez et al., 2010; Flowers, 2004; Kazachkova et al., 2018; Zhu, 2000). However, these two extremes in the plant salt‐tolerance spectrum represent less than 2% of all angiosperm diversity. Therefore, it is unclear how plants with varying degrees of salt‐stress responses growing in diverse salinity conditions conform to general expectations of how sodium accumulates in plants and how this accumulation affects their growth.

We conducted a systematic review of 49 published studies that included 67 species and 107 cultivars or populations, to identify broad‐scale patterns of salt accumulation and growth responses across terrestrial angiosperms. Employing a priori response models that we could test against experimental data, we surveyed the relationships between plant biomass growth and substrate NaCl concentration from controlled experiments across taxa. We also characterized relationships between plant‐tissue sodium accumulation and substrate NaCl concentration across taxa and examined how biomass growth responses associate with sodium accumulation. Finally, we assessed phylogenetic patterning of growth and sodium accumulation responses to reveal the role that evolutionary history has played in the distribution of these traits.

## 2 MATERIALS AND METHODS

### 2.1 Article search and selection protocol

To determine the effects of experimentally controlled, laboratory‐ or greenhouse‐based substrate sodium chloride (NaCl) treatments on plant biomass and sodium accumulation in their tissues, we searched for peer‐reviewed studies using Web of Science in December 2017 and May 2019 following the PRISMA protocol (Moher et al., 2009). We performed an initial search in December 2017 using the search criteria: “sodium AND biomass AND plant AND growth;” a timespan of “All years;” and indexes “Sci Expanded.” These criteria yielded 6,503 articles. For a second search in May 2019, we used the keywords: “sodium AND biomass AND plant OR sodium AND growth AND plant OR sodium accumulation AND shoot AND root AND plant OR sodium AND plant AND halophytes AND biomass;” a timespan of “All years;” and indexes “Sci Expanded.” This search yielded 6,654 articles. Subsequently, 6,387 duplicates were removed from the dataset, which produced a total of 6,770 non‐duplicate articles from the two searches.

The articles grouped into five unique categories: effects of sodium on growth, biomass, and tissue sodium accumulation in plants (1,305); salt‐related responses involving other taxa (animals, fungi, bacteria, protists, etc.) (906); transcriptomics, genomics, proteomics, or other molecular responses (627); influences of other elements and/or compounds (1,750); and other miscellaneous articles (2,183). We used the 1,305 articles that provided data for growth (biomass accumulation) and sodium accumulation in plant tissues.

In plants, biomass or biomass growth are often used as proxies for fitness, because they are often highly correlated with plant fecundity and survivorship. In addition, these fitness metrics can be easily applied across taxa to answer comparable questions across multiple species (Younginger et al., 2017). To investigate the relationship between substrate sodium and biomass changes, we retained 49 studies that reported aboveground and belowground dry biomass as well as aboveground and belowground sodium tissue concentration for a total of 107 cultivars, strains, or varieties (herein populations) of plants, in 67 species, 43 genera, and 20 families, across 16 orders (Table S1) (Abdallah et al., 2016, Al Sherif, 2009, Ashraf and Ahmad, 2000, Ashraf et al., 2001, Assaha et al., 2013, Barhoumi et al., 2007, Bayuelo‐Jiménez et al., 2003, Ben Hamed et al., 2014, Chartzoulakis et al., 1995, Ebrahimi and Bhatla, 2011, Ferreira et al., 2001, Gebauer et al., 2004, Gorai et al., 2007, Gul et al., 2010, Gulzar et al., 2003, Hamilton et al., 2001, Kafi and Rahimi, 2011, Kchaou et al., 2010, Keling and Zhujun, 2010, Khan et al., 2001, Khan et al., 2000a, Khan et al., 2000b, Khan et al., 2000c, Kim et al., 2012, Manivannan et al., 2008, Moghaieb et al., 2001, Mori et al., 2006, Naidoo, 1994, Nedjimi, 2009, Nedjimi, 2014, Parida et al., 2016, Qureshi et al., 2007, Rejili et al., 2007, Renault et al., 2001, Ruiz et al., 1997, Sanadhya et al., 2015, Shaheen et al., 2013, Shereen et al., 2007, Sohail et al., 2009, Taffouo et al., 2010, Tammam et al., 2008, Tounsi et al., 2017, Turan et al., 2010, Veatch‐Blohm et al., 2014, Waheed et al., 2006, Wu et al., 2015, Yokaş et al., 2008, Yue et al., 2012, Zouhaier et al., 2015).

Although these controlled experiments were conducted by different groups, in different controlled environments, and at different time scales, each used specific NaCl treatments between control and salt‐treated plants for a uniform duration specific to each study, keeping all other macronutrients and micronutrients constant. The plant material subjected to NaCl treatments was mostly seedlings (80.37%), with the remaining studies conducted on cuttings (13.08%), rootstocks/grafts (3.74%), and bulbs (2.80%). Prior to analysis, we updated nomenclatural changes for all species considered in this study using Tropicos (www.tropicos.org) and NCBI taxonomical databases (Table S2).

### 2.2 Data extraction and compilation

Articles differed substantially in their data representation, ranging from tables to graphical illustrations. We directly extracted data from tables, whereas measurements in figures were extracted using WebPlotDigitizer (Rohatgi, 2019). Treatments of NaCl were converted when necessary to mM. We focused on the mean responses of plants across treatments compared to their relevant control group as defined in each published study.

For biomass growth of aboveground (B_A_), belowground (B_B_), or total dry mass (B_T_), we extracted and converted, when necessary, all measurements in grams. Above and belowground biomass summed together equaled total plant biomass. We calculated relative biomass difference (RBD) for aboveground, belowground, or total biomass as:

$$\text{RBD}=\frac{\text{Treatment biomass}}{\text{Control biomass}}‐1.$$

Values of RBD greater than zero mean that growth under the treatment condition exceeded the growth observed for control plants. A negative RBD indicates that growth slowed in the salt‐treated plants compared to the control plants. While we note that growth itself cannot be negative, negative RBD values may represent salt‐induced shedding of leaves or similar plant responses that may directly affect the total biomass of experimental plants. RBD values corresponding to their raw experimental values for each study are given in Table S3.

Using the same methods described above, we extracted sodium concentrations per dry mass of aboveground, belowground, or total tissues. It is important to note that some plants may have expelled sodium, by means of salt glands or other adaptations. Tissue sodium concentration was considered as reported by each study. Acceptable sodium concentration measurements included weight‐by‐weight basis (i.e. mg/g, mg/kg), molarity (i.e. µM, mM or *M* (mol/L)), molality (i.e. mol/g), percentage (%), or parts per million (ppm). We converted all measurements, when necessary, to percentage (%) values. Measurements of electrical conductivity (S/m or psu) were excluded because, unless stated, they do not necessarily reflect sodium concentrations accurately since conductivity results from multiple elemental ions (Carter & Gregorich, 2007). Aboveground (Na_A_) and belowground (Na_B_) tissue sodium concentrations (%) were used to calculate total plant sodium concentration (Na_T_, %) using the formula:

$${\text{Na}}_{T}=\left(\frac{B_{A}}{B_{T}}*{\text{Na}}_{A}\right)+\left(\frac{B_{B}}{B_{T}}*{\text{Na}}_{B}\right).$$

All extracted raw data for sodium accumulation have been organized in Table S4.

### 2.3 Model design, selection, and population classification

We postulated a set of a priori potential response models for both RBD (Table 1) and sodium accumulation (Table 2) as functions of substrate NaCl treatments. Each a priori model prediction was described by a mathematical function for the shape of the response curve. Three pairs of responses shared an underlying mathematical function. For growth (Table 1), the function for a straight line accounted for both linear increase and linear decrease models and the slope of the line was used to classify the respective response: positive slope indicated linear increase, and negative slope indicated linear decrease. Also, the quadratic function accounted for both hump‐shaped and nonlinear decrease models. For sodium accumulation (Table 2), the quadratic function accounted for hump‐shaped and nonlinear increase. In these quadratic‐function cases, we used the vertex value (*a*) to classify cases as hump‐shaped (when *a* was negative) or nonlinear decrease and nonlinear increase (when *a* was positive).

**TABLE 1:** A priori response predictions for relative biomass growth and models used to classify populations in plants exposed to increasing concentrations of NaCl in the substrate

Model ID	Equation	Classification	A priori representation	Criterion of classification	Total plant responses	Aboveground responses	Belowground responses	Biological significance
I	y=mx+b	Linear increase	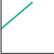	m	3 (2.8%)	3 (2.8%)	5 (4.7%)	Salt‐induced linear growth response
		Linear decrease	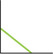	‐m	40 (37.4%)	34 (31.8%)	31 (29%)	Salt‐sensitive linear decrease in relative growth
II	$y=‐e^{x}$	Threshold decline	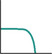	−*e*	11 (10.3%)	11 (10.3)	15 (14%)	Salt‐insensitive growth at lower sodium concentrations changed to rapid growth inhibition as external sodium increases
III	$y=ax^{2}+bx+c$	Hump‐shaped	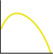	‐a	18 (16.8%)	18 (16.8%)	17 (15.9%)	Salt‐induced growth enhancement switches to growth inhibition as external sodium increases
		Nonlinear decrease	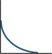	a	32 (29.9%)	33 (30.8%)	29 (27.1%)	Decelerating growth inhibition in response to increasing substrate salt
IV	y=b	Zero slope	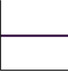		3 (2.8%)	3 (2.8%)	5 (4.7%)	Salt‐insensitive growth

**TABLE 2:** A priori response predictions for sodium accumulation responses and models used to classify populations in plants exposed to increasing concentrations of NaCl in the substrate

Model ID	Equation	Classification	A priori representation	Criterion of classification	Total plant responses	Aboveground responses	Belowground responses	Biological significance
I	y=mx+b	Linear increase	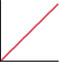		35 (32.7%)	39 (36.4%)	35 (32.7%)	Plants steadily and monotonically increase accumulation of sodium as sodium in the substrate increases
II	$y=e^{x}$	Exponential increase	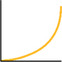	*e*	13 (12.1%)	12 (11.2%)	11 (10.3%)	Monotonic exponential increase in accumulation of sodium as sodium in the substrate increases
III	$y=ax^{2}+bx+c$	Hump‐shaped	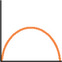	‐a	12 (11.2%)	5 (4.7%)	14 (13.1%)	Accumulation of sodium increases to a maximum and then decreases as sodium in the substrate increases; this is a non‐monotonic change, since the directionality of change reverses
		Nonlinear increase	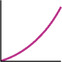	a	7 (6.5%)	5 (4.7%)	6 (5.6%)	Monotonic increase in accumulation of sodium is nonlinear as sodium in the substrate increases
IV	$y=a‐be^{‐cx}$	Asymptotic increase	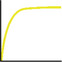		22 (20.6%)	24 (22.4%)	20 (18.7%)	Monotonic increase in accumulation of sodium at a decreasing rate, which then either approaches saturation or reaches a plateau, as sodium in the substrate increases
V	$y=\frac{1}{1+e^{‐x}}$	Sigmoidal increase	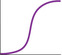		17 (15.9%)	16 (15%)	14 (13.1%)	Monotonic increase in accumulation of sodium is sigmoidal as sodium in the substrate increases
VI	y=b	Zero slope	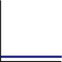		1 (0.9%)	1 (0.9%)	2 (1.9%)	Accumulation of sodium is unaffected by sodium in the substrate

We used an Information Criterion (IC) approach to select the model that best fit the data extracted for each population, using three different ICs: Akaike Information Criterion (AIC), the AIC small‐sample corrected version (AICc), and Bayesian Information Criterion (BIC). We used the R package ‘*AICcmodavg*’ to calculate AIC, AICc, and BIC values (Mazerolle, 2020). Although we examined results from all three metrics, we based our conclusions on BIC, since this metric gave consistent results across the data sampled, it is more specific (reduced Type‐I error or lower false‐positive rate), and it is considered a more conservative test, as advocated by Dziak et al. (2020). AIC is mainly recommended for larger datasets and does not account for sample size. Furthermore, for AICc, the penalization that is given to the AIC formula increases the chances of overfitting the data due to the extremely small sample sizes for the data analyzed (Bolker, 2008; Dziak et al., 2020). The models from Tables 1 and 2 that best fit each response (i.e. the smallest BIC value) were used to designate a response shape for each population's aboveground, belowground, and total plant biomass growth and sodium accumulation, respectively. Since we based our conclusions on BIC, we provide the corresponding likelihood values, ΔBIC and BIC weights for each model chosen; we also share results from the other two IC metrics for comparison (Tables S5–S8).

Fisher's exact test contingency analysis with simulated *p*‐values in R‐Studio following recommendations from Broman and Caffo (2003) was used to test for significant differences between growth and sodium accumulation. This test assumes that each population can be treated independently. This assumption may not be valid if the responses in certain groups are dependent on phylogenetic relationships (see next section for our analyses to test for such a bias).

To determine whether sodium accumulation differed by growth responses between aboveground and belowground tissues, for each growth response category we performed a Wilcoxon Test for paired values of aboveground versus belowground tissue sodium concentrations. For this test, we divided treatments into nonsaline (0 mM treatment of NaCl) and saline treatments (30–300 mM treatment of NaCl). For the saline group, the highest treatment concentration for each population was selected within the treatment range of 30–300 mM of NaCl.

### 2.4 Phylogenetic patterns among responses

We performed a phylogenetic signal analysis to assess whether phylogenetic relationships may have influenced growth and sodium accumulation responses in the diverse set of taxa used in our systematic review. The phylogenetic signal is the tendency of closely related species to resemble each other more in trait values than species drawn at random (Blomberg et al., 2003; Münkemüller et al., 2012). We used a subset of the rooted and dated ALLMB phylogeny from Smith and Brown (2018) for our phylogenetic signal analyses; this phylogeny consists of a backbone from Magallón et al. (2015) and data from both GenBank and the Open Tree of Life (Smith & Brown, 2018; available from https://github.com/FePhyFoFum/big_seed_plant_trees; Table S2). The phylogenetic tree of angiosperms was pruned using the ‘*drop.tip’* function from the *ape* package (Paradis & Schliep, 2019; v.5.3) to represent the species relevant to this study. In four cases (*Citrus sinensis, Solanum nigrum, Triglochin bulbosa,* and *Tripleurospermum maritimum*), subspecies were used as proxies in the phylogeny. For the genus *Narcissus*, we used the species *N*. *tazetta* for tree pruning (LoPresti et al., 2019). Additionally, for species that had multiple populations represented in our response dataset, we averaged population responses and selected the best models that fit the extracted data to assign overall responses for growth and sodium accumulation for each species (*Aeloropus lagopoides, Beta vulgaris, Brassica rapa, Cajanus cajan, Eucalyptus camaldulensis, Gossypium hirsutum, Helianthus annuus, Lotus creticus, Narcissus, Olea europaea, Oryza sativa, Phaseolus vulgaris, Solanum lycopersicum,* and *Solanum melongena*). A polytomy at the node for *Citrus* was resolved using the *phytools* package (Revell, 2012) function ‘*resolveNode’* and ‘*multi2di’* function from the *ape* package (Paradis & Schliep, 2019).

We tested for phylogenetic signals for the discrete characters of aboveground, belowground, and total plant growth and sodium accumulation responses using the Maddison and Slatkin (1991) method in the ‘*phylo.signal.disc’* function from Bush et al. (2016). This method estimates the minimum trait transitions at each node and compares this to a distribution sampled from a null model (Head et al., 2018; Paleo‐López et al., 2016). We used 1,000 randomizations to infer a significant result if the number of observed trait changes was significantly (*α* = 0.05) less than the median of the null model distribution. All data were analyzed using R software version 3.6.3 (R Core Team, 2020).

## 3 RESULTS

### 3.1 Increasing substrate NaCl has varied effects on total plant growth responses

**FIGURE 1:**
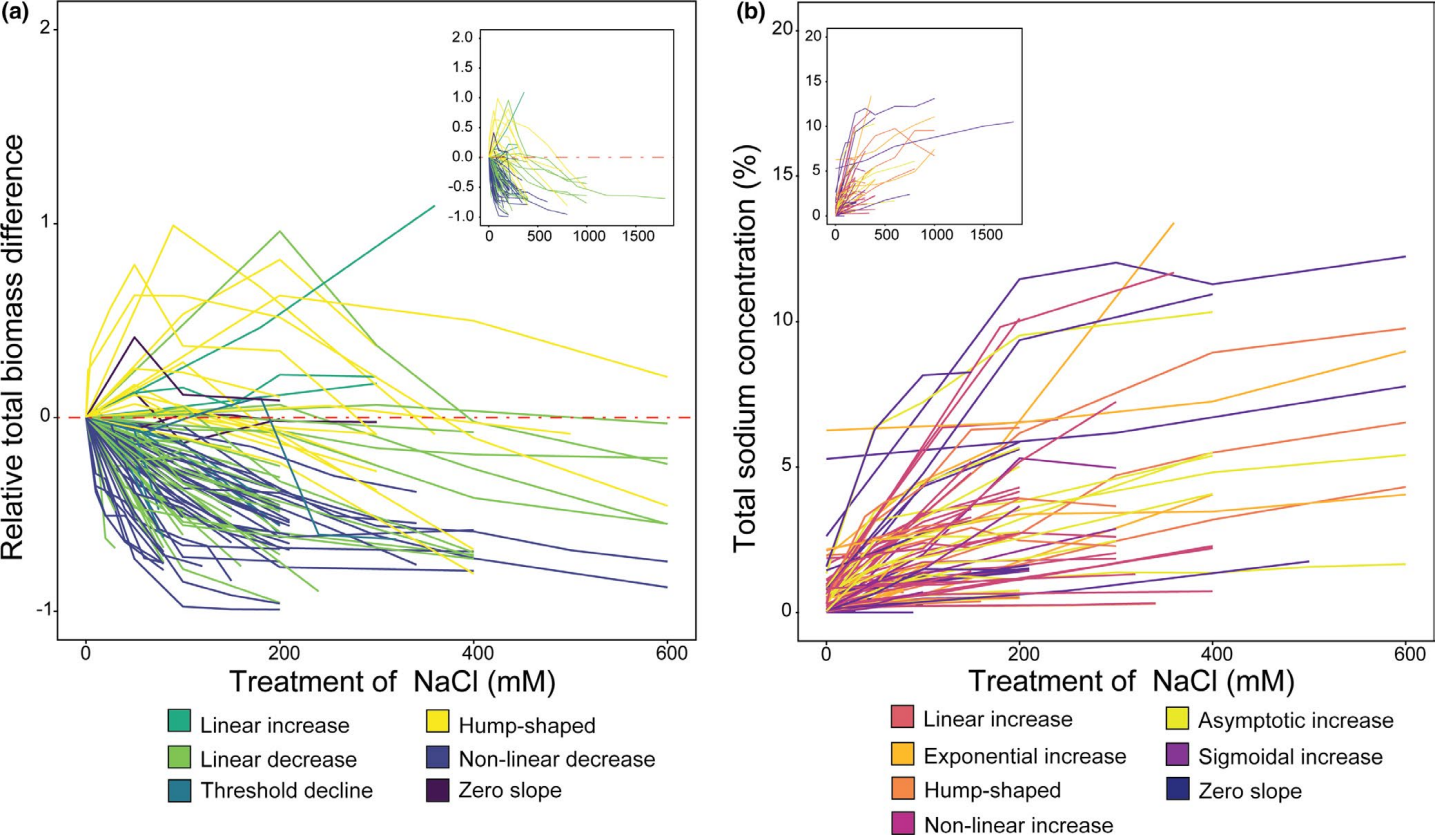
Populations' responses to increasing substrate NaCl concentrations. Total relative biomass growth responses (a) across NaCl treatments for each population sampled in the study. Negative and positive values represent a growth inhibition or an increase, respectively, in growth relative to control NaCl substrate concentrations. Also, the effect of NaCl treatments on total plant sodium accumulation (b) across increasing NaCl substrate concentrations for each population. The main data shown cover the range from 0 to 600 mM treatments of NaCl. An inset with the complete dataset and treatments is included with each panel. Colors represent the responses that describe biomass growth and sodium accumulation responses, as in Tables 1 and 2

Using model selection for each of our chosen 107 populations, we classified relative total plant growth responses as shown in Table 1 (Table S1). Growth was negatively affected as sodium increased in the substrate for most taxa. However, 12 taxa grew better in at least one treatment ≥200 mM NaCl. Growth was severely reduced in all populations that were exposed to NaCl concentrations >500 mM as compared to 0 mM of NaCl (Figure 1). None of the populations that we classified as having linear increase or zero slope biomass responses were exposed to treatments >360 mM NaCl.

**FIGURE 2:**
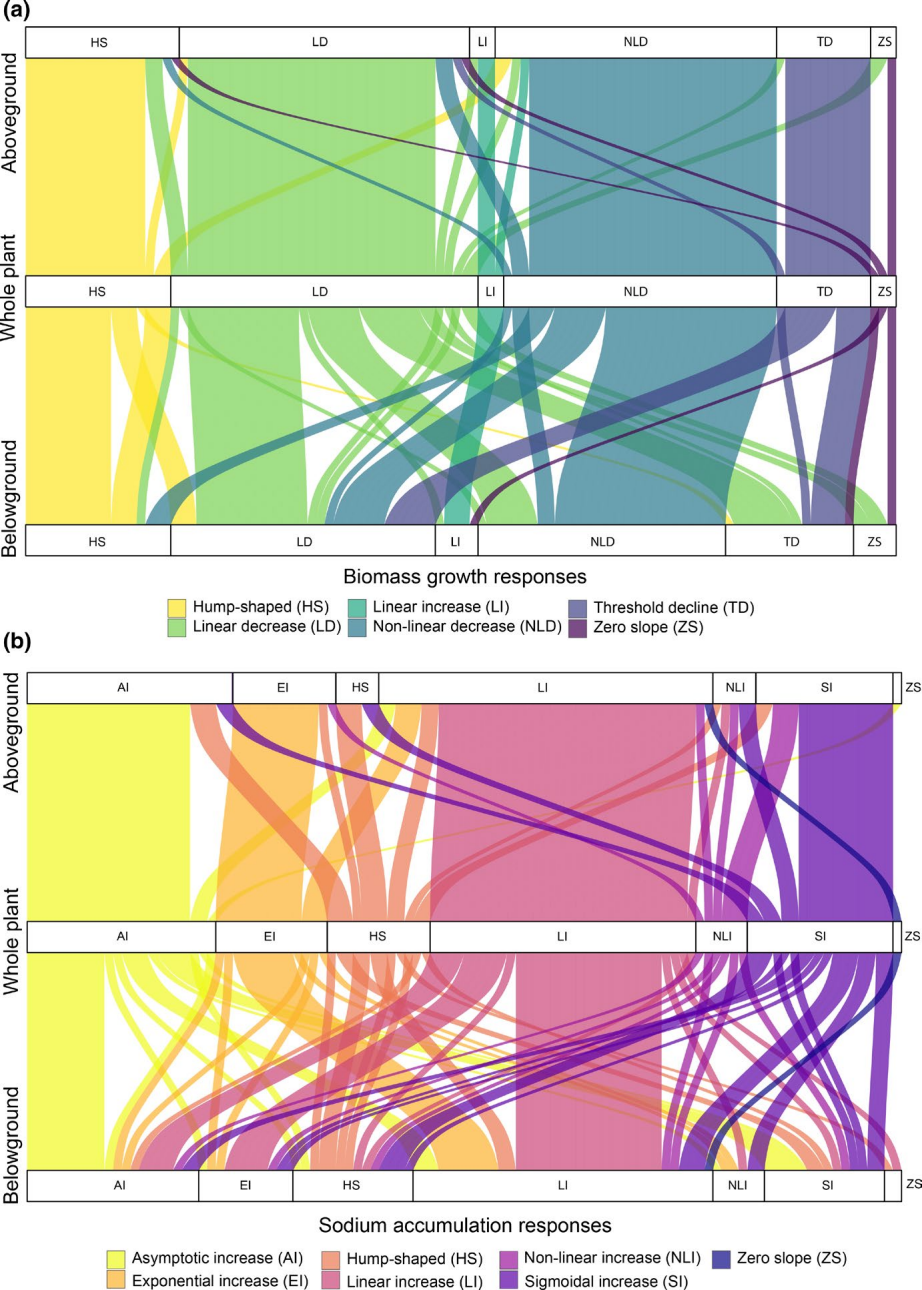
Alluvial plot describing the association between above‐and belowground phenotype responses to total plant biomass (a) and sodium accumulation (b). Thickness of each connector indicates the proportion of populations in each response group

Plant growth based on relative biomass difference showed similar trends in response to increased salinity regardless of the tissue sampled from aboveground or belowground (Figure S1a,b). The overall growth patterns of aboveground or belowground tissue mirrored the patterns observed at the total plant level, as visualized by similarity in the alluvial plot (Figure 2a).

### 3.2 Total plant sodium increases as substrate sodium increases

Using model selection for each of the 107 populations, we classified total plant sodium accumulation responses into six groups shown in Table 2 (Table S1). The total sodium concentration within a plant generally increased as the substrate concentration of sodium increased (Figure 1b). However, the level of sodium accumulation was highly variable among populations and between aboveground and belowground tissues (Figure S1c,d). Notably, the aboveground sodium concentrations were generally higher than in belowground tissues for most populations in saline treatments (Figure S1c,d). Additionally, regardless of the variation observed, both relative aboveground and belowground responses were similar to relative total sodium accumulation responses (Figure 2b).

### 3.3 Crop species do not adequately represent general plant responses

**FIGURE 3:**
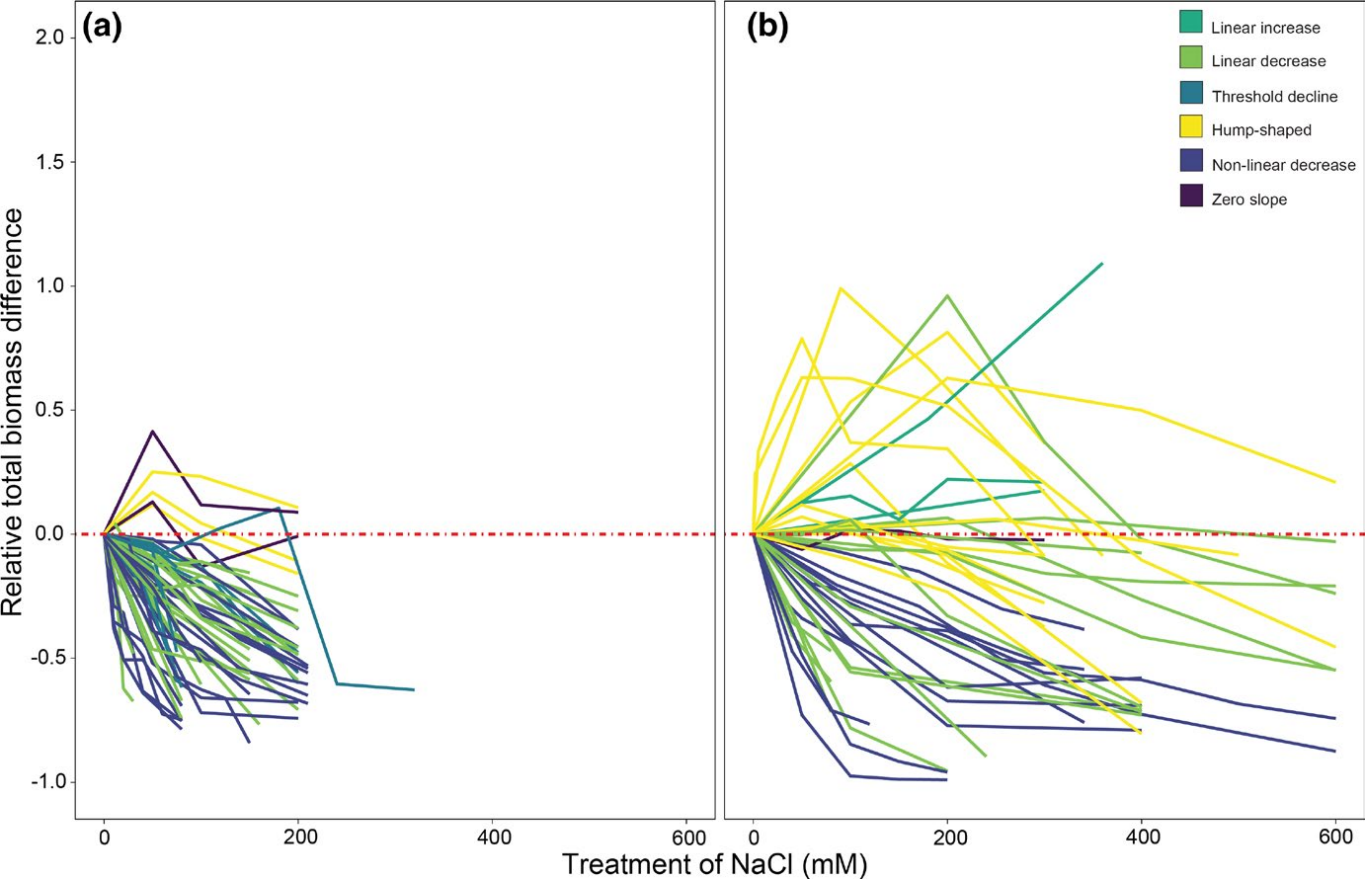
Growth responses to increasing substrate NaCl for (a) crop and (b) non‐crop populations

**FIGURE 4:**
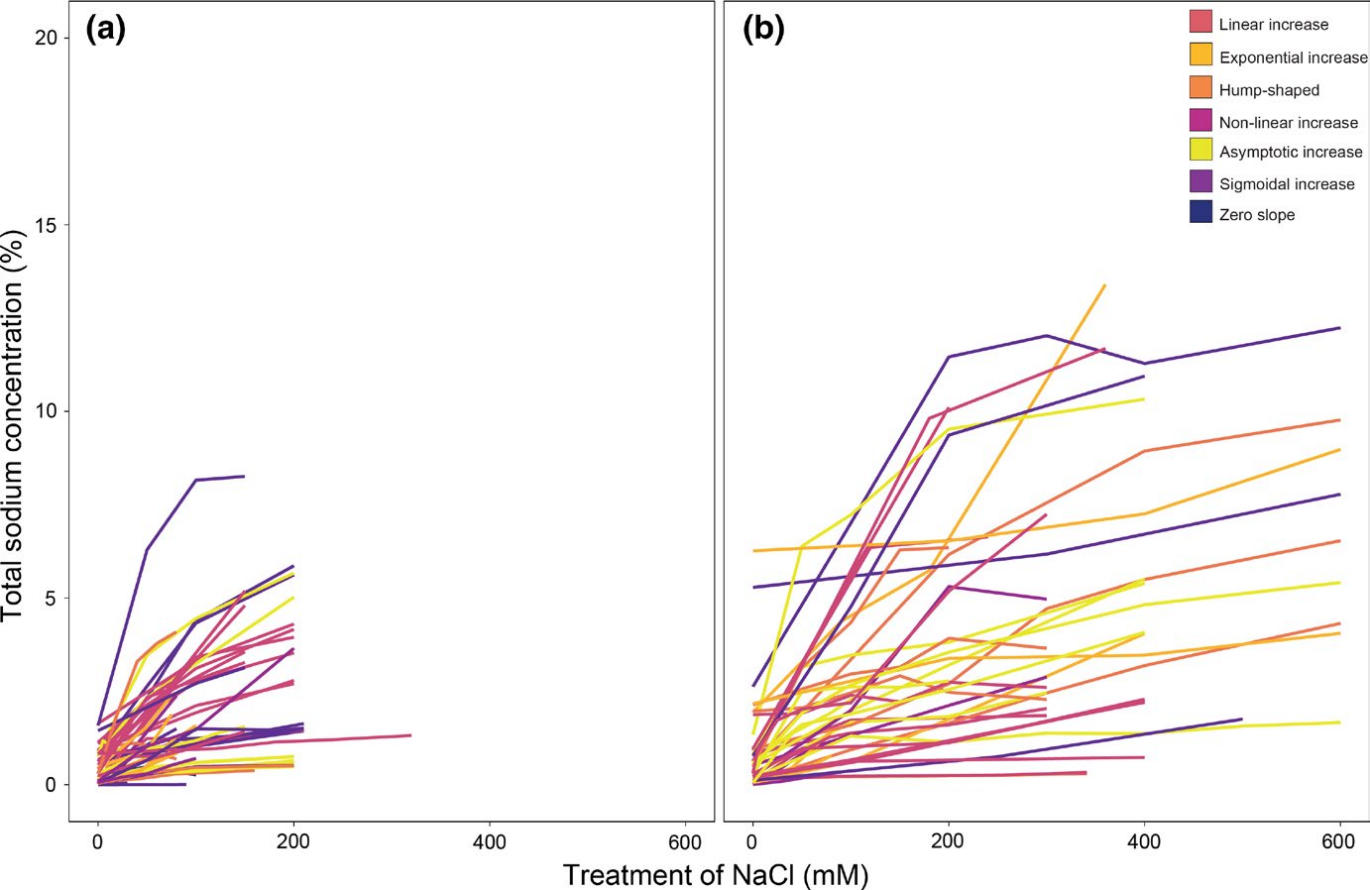
Sodium accumulation responses to increasing substrate NaCl for (a) crop and (b) non‐crop populations

In our study, crop species represented 43.3% (29) with only seven of them including populations surpassing 200 mM experimental exposure to substrate NaCl (Figure 3). Growth responses were generally more variable in non‐crop populations, with hump‐shaped growth responses being more prominent in non‐crop (26.7%) than crop (4.8%) populations (Figure 3a). Moreover, percent differences in tissue sodium concentration varied more in non‐crop than crop populations (i.e. variability in tissue sodium concentration was higher in non‐crop taxa) (Figure 4).

### 3.4 Plant growth responses do not predict sodium accumulation responses

**FIGURE 5:**
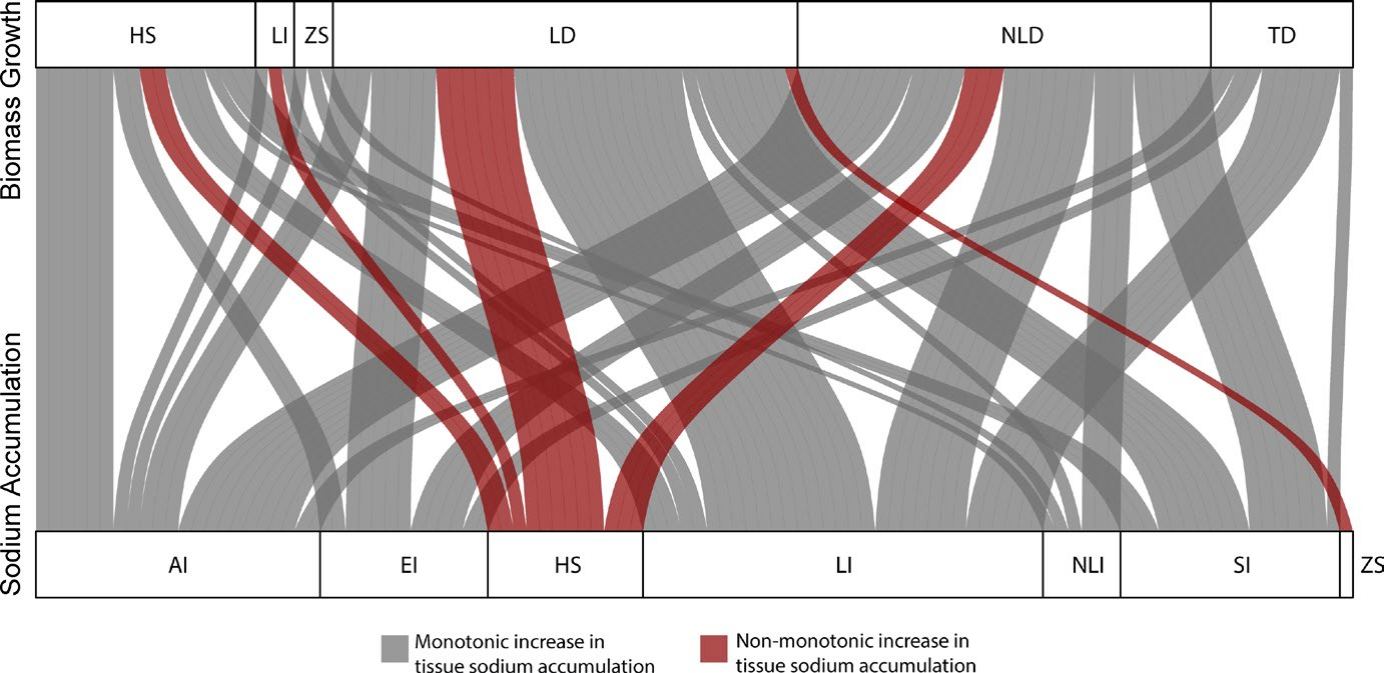
Alluvial plot describing the associations between biomass growth and sodium accumulation responses. Sodium accumulation responses were either monotonically increasing (grey) or not (maroon). Thickness of each connector indicates the proportion of populations in each response group. Responses for growth where abbreviated as follows: Hump‐shape (HS), linear decrease (LD), linear increase (LI), nonlinear decrease (NLD), threshold decline (TD) and zero slope (ZS). For sodium accumulation responses were abbreviated as follows: Asymptotic increase (AS), exponential increase (EI), hump‐shaped (HS), linear increase (LI), non‐linear increase (NLI), sigmoidal increase (SI) and zero slope (ZS)

Total plant biomass growth responses were largely independent of the type of sodium accumulation response, which we illustrate using an alluvial plot (*p* = .43; Figure 5). Furthermore, irrespective of the growth response, tissue sodium concentrations increased monotonically in the majority of populations, i.e. increase in plant sodium continued at a steady positive rate as sodium in the substrate increased or increased to a plateau for 77% of the populations (Figures 1 and 5).

**FIGURE 6:**
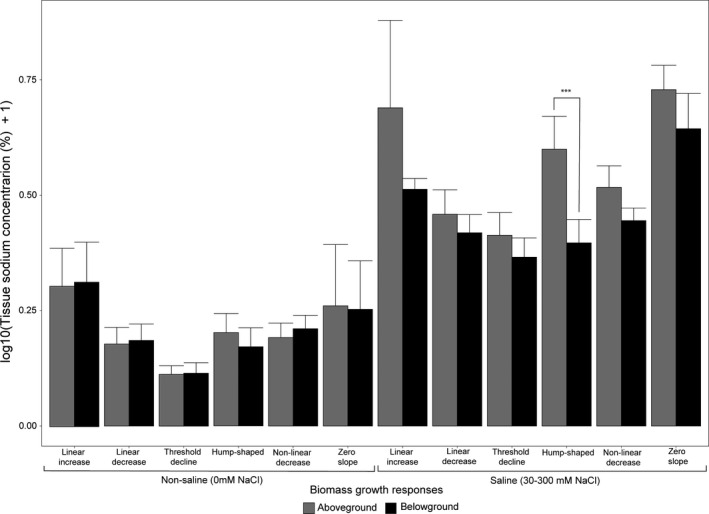
Mean log‐transformed tissue sodium concentration (%) (and SE) for above‐ and belowground tissues across biomass growth responses for non‐saline (0 mM NaCl) and saline treatments (30–300 mM NaCl). Significant differences (*p* < .001, Wilcoxon Test) for above‐ and belowground mean response comparisons are indicated by asterisks (***). Sample sizes for each growth response for above‐ and belowground tissues were the same for non‐saline and saline treatments: hump‐shaped = 17; linear decrease = 36; linear increase = 3; non‐linear decrease = 32; threshold decline = 11; and zero slope = 3

Only those populations with hump‐shaped growth responses differed significantly in sodium accumulation between aboveground and belowground tissues across saline treatments (Wilcoxon Test: *n* = 17, *Z* = 1.9, *p* > .046). There were no statistically significant differences for any other biomass growth responses between sodium accumulation of aboveground versus belowground tissues across saline treatments. Additionally, for nonsaline treatments, there was no statistically significant difference for any biomass growth response groups when aboveground and belowground sodium accumulation was compared (Figure 6).

### 3.5 Phylogenetic relationships predict biomass growth but not sodium accumulation responses

**FIGURE 7:**
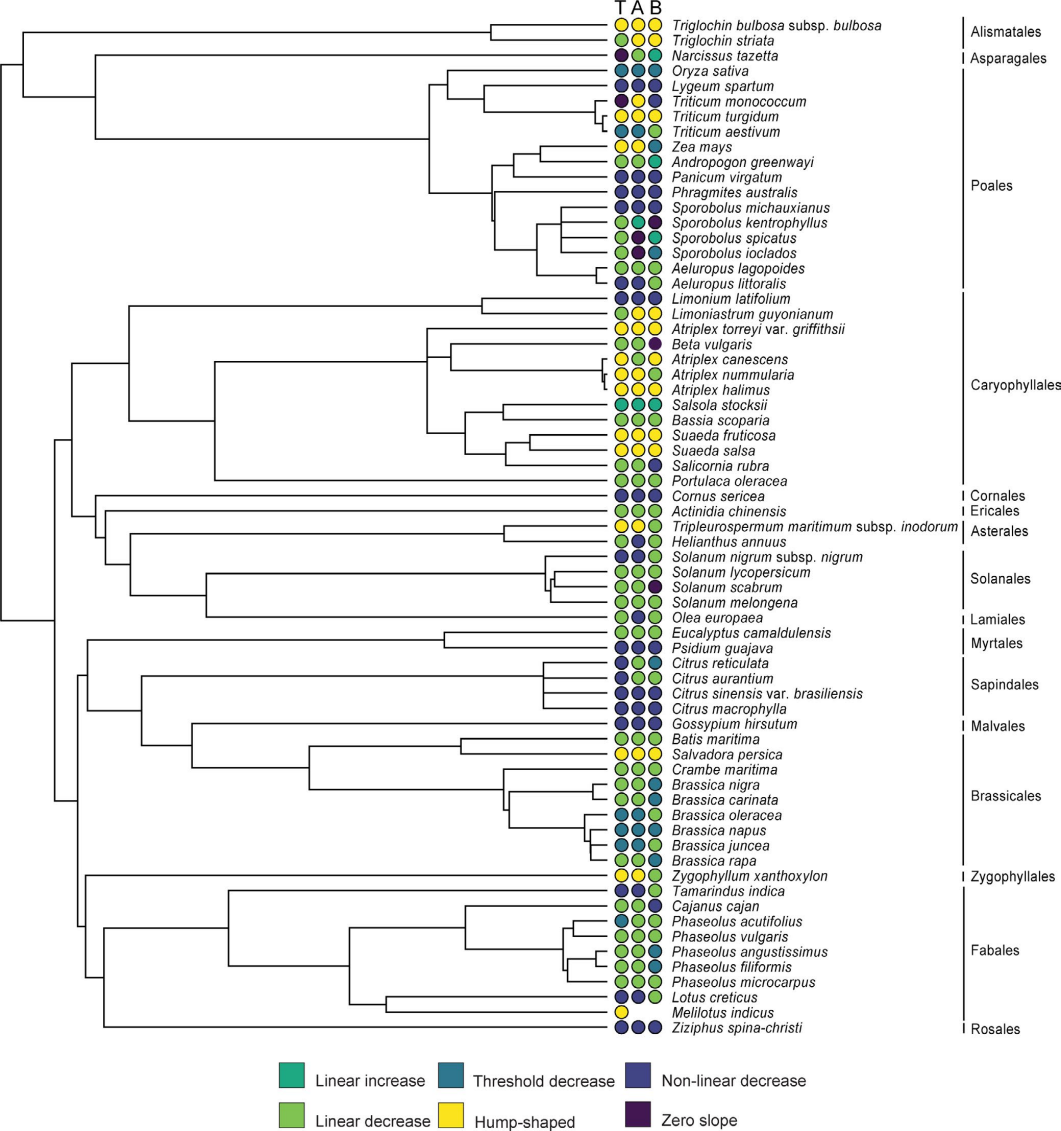
Total (T), above‐ (A) and belowground (B) plant biomass growth responses mapped onto a phylogeny. Tips represent species pruned from rooted and dated ALLMB phylogeny from Smith and Brown (2018). Plant orders are indicated to the right of the phylogeny

Biomass growth, both aboveground and belowground, showed significant phylogenetic signal (i.e., phylogenetic relationships help explain the distribution of the trait across the phylogenetic tree in our dataset; *p* = .031 and *p* = .046, respectively; Figure 7). We recovered 28 observed evolutionary transitions (i.e. the change from one discrete trait to another) with a randomization median of 35 for aboveground biomass growth response. Belowground biomass growth response showed 33 observed evolutionary transitions and a randomization median of 37 transitions. We found significant phylogenetic signal for total biomass response (*p* = .012) with 29 observed evolutionary transitions and 34 median randomization transitions. Most of the species in the order Caryophyllales, especially in the family Amaranthaceae, expressed a hump‐shaped biomass growth response as sodium increased in the substrate. However, hump‐shaped responses were also found in other plant orders, reflecting potential independent evolutionary origins.

**FIGURE 8:**
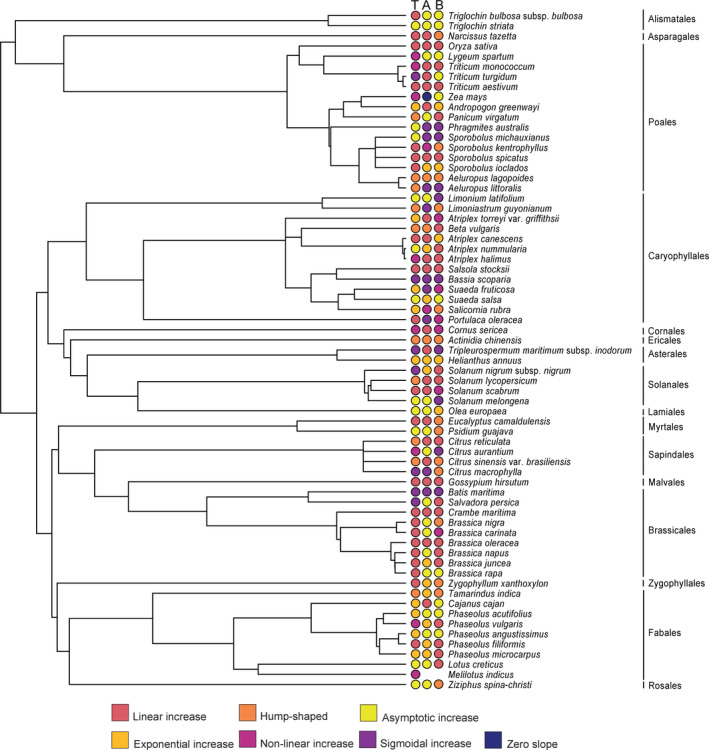
Total (T), above‐ (A) and belowground (B) plant sodium accumulation responses mapped onto a phylogeny. Tips represent species pruned from rooted and dated ALLMB phylogeny from Smith and Brown (2018). Plant orders are indicated to the right of the phylogeny

Sodium accumulation responses (both aboveground and belowground) were not significantly phylogenetically organized, that is, did not show significant phylogenetic signal (*p* = .37 and *p* = .184, respectively; Figure 8). For aboveground sodium accumulation response, there were 36 observed evolutionary transitions while the randomization median was 37. We found 35 observed evolutionary transitions and 37 randomized median transitions for belowground sodium accumulation response. No phylogenetic signal was found for total plant sodium accumulation response (*p* = .161), and we recovered 38 observed transitions with a randomized median of 40 transitions. For the orders most sampled, Caryophyllales and Poales, responses for sodium accumulation differed substantially across and within genera, with no apparent pattern observed. Plants appeared to accumulate sodium in different ways and patterns regardless of their biomass growth responses.

## 4 DISCUSSION

Understanding the influence of sodium in the substrate on plant performance (growth, fitness) and tissue sodium accumulation is essential to comprehend ecological and evolutionary dynamics of plants across terrestrial environments. Our study emphasizes that plant adaptations to substrate sodium vary substantially across taxa in terms of growth and sodium accumulation with a degree of phylogenetic conservatism. However, regardless of growth responses, sodium accumulation mostly followed an increasing trend and did not have any apparent association to growth responses as substrate sodium levels increased. Additionally, we note that for the taxa for which we had data, domesticated plant species present a narrower range of variation among salt stress responses as compared to non‐crop species. In order to understand how substrate sodium influences plant growth, we must consider non‐crop species in our studies. Moreover, we advocate the importance of characterizing responses using a systematic approach, and we provide recommendations on experimental designs to reach a broader understanding of plant‐salt stress.

### 4.1 Increasing substrate sodium influences plant growth and sodium accumulation in variable ways

Saline soils are known to hinder plant growth, and crop losses are reported when soil salinity is above a crop‐specific threshold (Bernstein, 1975; Zhao et al., 2020; Zörb et al., 2019). Whereas our analysis is aligned with this general consensus on the negative impact of soil salinity on plant growth, it sheds light on how plant growth varied in response to substrate NaCl levels across plant taxa that ranged from highly studied crops to scarcely examined wild species (Table 1 and Figure 2a,b). Despite the overall trend of decreased biomass concurrent to increasing substrate NaCl levels, several taxa in the order Caryophyllales (*e.g*., families Amaranthaceae, Plumbaginaceae and Portulacaceae) showed a hump‐shaped or linear increase in biomass growth to increasing substrate NaCl (Figures 1a and 7). Most halophytes are non‐randomly distributed, and the order Caryophyllales holds the greatest number of recorded halophytes among angiosperms (Flowers et al., 2010). Halophytes not only are tolerant of high NaCl, but also use Na^+^ and Cl^‐^ ions for osmotic adjustment in an energetically favorable manner and are equipped with structural and physiological traits that aid the compartmentalization of salts to promote growth while avoiding ionic or osmotic stress until threshold NaCl levels are reached (Munns et al., 2020; Slama et al., 2015). This set of characteristics would account for the positive growth in saline substrates that we observed within the Caryophyllales taxa (Figures 1a and 7). Furthermore, plants that follow these hump‐shaped or linear increase growth responses to increasing substrate sodium follow a subsidy‐stress gradient, i.e. at low substrate sodium levels overall plant growth is subsidized, reaching a threshold leading to growth inhibition due to salt stress as sodium in the substrate becomes toxic (Odum et al., 1979). All plants that followed these trajectories in our analyses (Figure 7) are considered salt tolerant, as classified in the eHALOPH database (Santos et al., 2016) and by the respective authors in each study (Table S1). Regardless, even among those salt‐tolerant taxa, plant biomass eventually decreased at the highest NaCl concentrations (Figure 1a). The use of sodium as an inexpensive osmolyte has convergently evolved in many halophytes as well as other plants adapted to water deficit stress and is found in multiple orders of plants. For example, even at low sodium levels in the soil, the xeric adapted plant, *Zygophyllum xanthoxylum* (Zygophyllaceae), accumulates high concentrations of sodium in shoots, resulting in large mesophyll cells leading to leaf succulence (Xi et al., 2018).

The taxa that showed linear or nonlinear decreases (Figures 1a and 7) as NaCl increased in the substrate are non‐halophytes highly sensitive to salt stress where growth is inhibited by excess salts (Munns et al., 2020; van Zelm et al., 2020). Moreover, we found that closely related lineages resembled each other with respect to biomass growth responses (i.e. significant phylogenetic signal indicating shared physiological responses within clades); thus, the patterns observed in this trait are at least somewhat explained by shared evolutionary history (Figure 7). However, phylogenetic patterns do not account for sodium accumulation responses (Figure 8).

In plants, tissue sodium concentrations are generally linked with increasing substrate sodium concentrations (Figure 1b). However, plant sodium accumulation seemed to be uncoupled from biomass growth responses and any discernible phylogenetic signal among taxa (Figures 5 and 8). Similar patterns were observed when aboveground sodium accumulation was compared in the species *Plantago maritima* and *Plantago media* as NaCl in the substrate was increased (Maathuis, 2014; note that these populations – among others in the literature – were not included in the current study since they did not meet the criteria for our selection). The variation in responses by each species was mainly due to differential and discrete tolerance thresholds and external sodium concentrations (Maathuis, 2014), which might explain the idiosyncratic variation that was observed among taxa used in this study in terms of sodium accumulation responses (Figures 1b and 8).

Additionally, the accumulation of higher amounts of sodium in aboveground (Figure S1c) than belowground (Figure S1d) tissues is apparent when comparing sodium accumulation responses for each population across increasing treatments of substrate NaCl (Figure 1b). This observation agrees with the current understanding that sodium, once in the transpiration stream, is retained in the shoots as phloem recirculation to roots and is considerably less then xylem loading from roots to shoots (Munns, 2002; Munns & Tester, 2008). Sodium accumulation in the shoots is dependent on the local tissue and species‐specific tolerance capacity. Plants are known to store excess sodium in older leaves to protect younger growing tissue from salt toxicity, and sustain growth until species‐specific tolerance levels are reached (Munns & Tester, 2008). Alternatively, a few halophytes have developed salt glands to remove sodium from shoots against a concentration gradient – an adaptation that is found in several plant orders (Dassanayake & Larkin, 2017).

Once sodium enters the roots, plants have transporters that preferentially export sodium back to the soil at an energy cost. However, this capacity to export sodium at the soil–root interphase is easily exceeded even among halophytes, and accumulation of sodium inside the plant is unavoidable when external sodium concentrations increase (Zhao et al., 2020). Therefore, other sodium transporters that facilitate ionic balance throughout the plant organs play critical roles in sustaining growth or survival during salt stress (Apse & Blumwald, 2007; Yamaguchi et al., 2013). Our systematic review agrees with previous studies investigating single or small groups of taxa subjected to salt stress to highlight that almost all plants accumulated sodium monotonically (or nearly monotonically) as sodium increased in the substrate (Figures 1b and 5). Plants that expressed the biomass growth hump‐shaped response accumulated significantly higher concentrations of sodium in aboveground than belowground tissues. Alternatively, populations characterized by the other growth responses did not differ significantly in aboveground versus belowground sodium accumulation in saline treatments but not in nonsaline treatments (Figure 6). We discussed earlier that the hump‐shaped response was preferentially represented by taxa in the order Caryophyllales and that this clade is an evolutionary hotspot for halophytes, but this response is not confined to the order (Figure 7). Furthermore, Caryophyllales species often are shoot sodium hyperaccumulators; they are enriched in plants that develop salt glands and have a higher tolerance to higher tissue sodium levels than predominantly salt‐sensitive orders (Dassanayake & Larkin, 2017; Flowers et al., 2010; White et al., 2017).

### 4.2 Domesticated plant species tend to occupy a narrow range of variation among salt stress responses

Our systematic review demonstrated a clear dichotomy between salt tolerance (deduced from growth responses) during increased external sodium in crops compared to wild species or plants that have not been subjected to domestication. Most wild species in our study tend to have a higher capacity to tolerate higher tissue sodium than crop or domesticated species (Figure 2a,b). The exception to this is seen with crops in Caryophyllales, such as *Beta vulgaris, Salicornia bigelovii,* and *Spinacia oleracea* (Choo et al., 2001; Wu et al., 2013; Yamada et al., 2016). Recent studies have illustrated how crop species have lost traits related to salt tolerance their ancestral wild relatives had before domestication (Quan et al., 2018; Rozema et al., 2015; Wang et al., 2020, 2021).

The individual studies used for our systematic review are limited to small and variable sample sizes among populations, differing treatment concentrations of NaCl, and include a mixture of crop (43.3%) and non‐crop (56.7%) species. Salt stress responses in plants are known to vary in how the salt treatment is given (acclimated treatment vs. salt shock), duration of the treatment, the age of the plants, plant growth conditions (e.g. light levels, presence of other stresses, and grown hydroponically or in soil, tidal systems, submerged systems), plant habit (e.g. herb vs. tree, creeper vs. upright), life history traits (e.g. annual vs. perennial, frequency of flowering), morphological traits of the plants (e.g. presence or absence of salt glands, ability to produce succulent leaves, structural adaptations in roots), among many other genetic and environmental factors (Polle & Chen, 2015; Zhao et al., 2020). Plant survival compared to growth may use different adaptive traits among plants, and biomass may not be the only indicator nor the optimal indicator to measure salt responses among different groups of plants. Therefore, systematic and rigorous studies need to be performed to understand overall mechanisms underlying salt stress responses across taxa, as discussed in the next sections.

### 4.3 Characterizing responses promotes our understanding of plant‐salt stress

The modeling approach that we used in this study provides a useful way to quantify and categorize individual plant population responses to variation in NaCl in the substrate. These models describe the response trajectories of biomass growth and sodium accumulation responses and could be used extensively across taxa of interest. By using an Information Criterion approach, one can select the best‐fit model for each population, given that our formulated models (e.g. linear decrease, hump‐shaped, etc.) effectively describe natural patterns (Brewer et al., 2016), within and among species (Tables 1 and 2). For many purposes, it may be more useful to categorize plants by their responses across a range of sodium conditions, as opposed to performance above and below strict thresholds, as is often done with halophytic or salt‐tolerant plants (see Grigore et al., (2014) for a review on definitions and descriptions related to halophytes).

### 4.4 Experimental design to achieve broader understanding

Many studies have tested the effects of NaCl on plant growth and yield, especially in crop species (Cheeseman, 2015). However, because of differences in methodology, it is a challenge to make comparisons and contrasts of results across studies. We make several observations and recommendations for future studies:

- Often, there is a lack of enough replication and/or treatments. For us, this prevented effective response pattern identification in some cases, especially in studies that presented only three treatments with few replicates.
- The determination of treatments was often arbitrary. Limitations are imposed using independent categorical variables (ANOVA‐based approach) instead of applying treatments as independent numeric discrete or continuous variables (regression‐based approach). Experimental designs that cover a wide range of treatments may provide more accurate estimates. A regression‐based approach allows one to better fit nonlinear responses, which encompasses most of the responses we measured in our study (Inouye, 2001; Whitlock & Schluter, 2014). Additionally, when resources are limited, experimental design should prioritize increasing the number of treatments over increasing number of replicates per treatment. Furthermore, functional growth analysis (i.e. the assessment of the absolute growth rate and relative growth rate) should be performed to better comprehend how plants manage resources at different life stages or across multiple environmental stresses, especially in the context of biomass growth and ionic accumulation (Cheeseman & Wickens, 1986; Tessmer et al., 2013).
- Most of the plants in the studies selected were not exposed to the highest levels of sodium they could potentially encounter in nature. Lack of these data thwarts the complete description of responses associated with increasing substrate NaCl within and across taxa. Linear increase responses are highly unlikely across all NaCl concentrations observed in nature. This type of response in our study likely results from lack of high NaCl treatments. Under the full range of NaCl, these taxa would most likely have hump‐shaped responses. Additionally, we observed that in nonsaline treatments (0 mM substrate NaCl), substantially large amounts of sodium were found in some plant taxa. The reason for this could have been the lack of attention to the ionic salts used in the Hoagland solution; some salts are combined with sodium (i.e. EDTA, Na_2_MoO_4_ 2H_2_O, etc.). Another reason could be the use of tap water instead of distilled or deionized water. Generally, a combination of copper, calcium, magnesium, and sodium is found in tap water on average at 1%, with some regional variation (Patterson et al., 2013).
- Many of the plants in the studies selected were grown under controlled conditions using watering regimes and nutrient mixes that do not closely reflect conditions in nature. Future research should focus on plant morphological, physiological, and adaptive responses to treatment solutions and/or substrates that truly match conditions (water availability, nutrient stoichiometry, etc.) potentially found in nature.
- Studies generally focus on biomass to the exclusion of other fitness‐related traits. Even though biomass is often an acceptable proxy for fitness measurements in plants (Younginger et al., 2017), observations on flower production, survivorship, seed set, and seed germination success should be quantified, to provide a more complete understanding of sodium's influence on whole‐plant performance and fitness (Primack & Kang, 1989).
- Studies also should consider that salt stress is often combined with water deficit and heat stress, or other nutrient stresses in natural habitats. Additionally, biotic stresses such as herbivory and disease can compound the overall plant response to salt stress, with special consideration of wild taxa. The net outcome of plant performance under these natural conditions needs to be assessed and compared to responses observed under controlled environments to be able to model plant responses at community or ecosystem scales.

### 4.5 Moving toward an ecological‐evolutionary perspective: from the lab to the field

We focused on plant performance and sodium accumulation strategies in controlled settings as reported in the literature, which emphasizes the physiological aspects of substrate sodium rather than the ecological and selective effects of sodium on plant performance, including fitness, under environmental conditions in nature. More importantly, this systematic review suggests support for the general No‐Escape‐From‐Sodium Hypothesis, i.e. that generally plants' tissue sodium levels reflect (at least in a ranked fashion) substrate/solution sodium levels irrespective of their growth responses to sodium (potentially with key and interesting exceptions). We still have a long way to go to be able to fully test this hypothesis, especially under the natural field conditions that truly matter for plant evolution, ecology, and farming.

Moreover, assessments of the phytochemical landscape of sodium across large geographical areas is increasing, with examples in *Ficus* in Central and South America (Bravo & Harms, 2017), *Asclepias* (milkweeds) in Minnesota (Mitchell et al., 2020), among roadside plant communities in Massachusetts (Bryson & Barker, 2002) and across global grasslands (Borer et al., 2019). These examples demonstrate that aboveground plant sodium accumulation co‐varies closely with some abiotic factors, including but not limited to effective distance from nearest coast/saline habitat; road salt pollution; and concentration of sodium in the soil. However, experimental designs that include comprehensive plant growth metadata, phenotyping, and careful selection of target plants to allow rigorous, yet broad comparisons are needed. Following our recommendations would help advance our understanding of the complexity of the formation of the phytochemical landscape of sodium and its ecological and evolutionary consequences for plant performance, sodium accumulation, and plant–herbivore interactions. In any case, saltier plants in saltier soils are proving to be a broadly general pattern for sodium, which begs the future research question: how do plants respond to all of the other elements in their substrates?

## CONFLICT OF INTEREST

The authors have no competing interest to declare.

## AUTHOR CONTRIBUTIONS

**Luis Y. Santiago‐Rosario:** Conceptualization (lead); Data curation (lead); Formal analysis (lead); Methodology (lead); Visualization (lead); Writing‐original draft (lead); Writing‐review & editing (equal). **Kyle E. Harms:** Conceptualization (equal); Supervision (lead); Writing‐original draft (equal); Writing‐review & editing (equal). **Bret D. Elderd:** Formal analysis (equal); Methodology (equal); Supervision (equal); Writing‐review & editing (equal). **Pamela B. Hart:** Formal analysis (equal); Methodology (equal); Visualization (equal); Writing‐review & editing (equal). **Maheshi Dassanayake:** Conceptualization (equal); Writing‐original draft (equal); Writing‐review & editing (equal).

## Data Availability

Data and additional supplementary information are available in https://doi.org/10.5061/dryad.h44j0zpk1.
